# Quantitative Blood Oxygenation Level Dependent Magnetic Resonance Imaging for Estimating Intra-renal Oxygen Availability Demonstrates Kidneys Are Hypoxemic in Human CKD

**DOI:** 10.1016/j.ekir.2023.02.1092

**Published:** 2023-03-07

**Authors:** Pottumarthi V. Prasad, Lu-Ping Li, Bradley Hack, Nondas Leloudas, Stuart M. Sprague

**Affiliations:** 1 Department of Radiology, NorthShore University HealthSystem, Evanston, Illinois, USA; 2 Department of Medicine, NorthShore University HealthSystem, Evanston, Illinois, USA; 3 Pritzker School of Medicine, University of Chicago, Chicago, Illinois, USA

**Keywords:** BOLD, CKD, kidney, MRI oxygenation

## Abstract

**Introduction:**

Kidney blood oxygenation level dependent (BOLD) magnetic resonance imaging (MRI) has shown great promise in evaluating relative oxygen availability. This method is quite efficacious in evaluating acute responses to physiological and pharmacologic maneuvers. Its outcome parameter, R2∗ is defined as the apparent spin-spin relaxation rate measured in the presence of magnetic susceptibility differences and it is measured using gradient echo MRI. Although associations between R2∗ and renal function decline have been described, it remains uncertain to what extent R2∗ is a true reflection of tissue oxygenation. This is primarily because of not taking into account the confounding factors, especially fractional blood volume (fBV) in tissue.

**Methods:**

This case-control study included 7 healthy controls and 6 patients with diabetes and chronic kidney disease (CKD). Using data before and after administration of ferumoxytol, a blood pool MRI contrast media, the fBVs in kidney cortex and medulla were measured.

**Results:**

This pilot study independently measured fBV in kidney cortex (0.23 ± 0.03 vs. 0.17 ± 0.03) and medulla (0.36 ± 0.08 vs. 0.25 ± 0.03) in a small number of healthy controls (*n* = 7) versus CKD (*n* = 6). These were then combined with BOLD MRI measurements to estimate oxygen saturation of hemoglobin (StO_2_) (0.87 ± 0.03 vs. 0.72 ± 0.10 in cortex; 0.82 ± 0.05 vs. 0.72 ± 0.06 in medulla) and partial pressure of oxygen in blood (bloodPO_2_) (55.4 ± 6.5 vs. 38.4 ± 7.6 mm Hg in cortex; 48.4 ± 6.2 vs. 38.1 ± 4.5 mm Hg in medulla) in control versus CKD. The results for the first time demonstrate that cortex is normoxemic in controls and moderately hypoxemic in CKD. In the medulla, it is mildly hypoxemic in controls and moderately hypoxemic in CKD. Whereas fBV, StO_2_, and bloodPO_2_ were strongly associated with estimated glomerular filtration rate (eGFR), R2∗ was not.

**Conclusion:**

Our results support the feasibility of quantitatively assessing oxygen availability using noninvasive quantitative BOLD MRI that could be translated to the clinic.

## Graphical abstract

**Figure:**
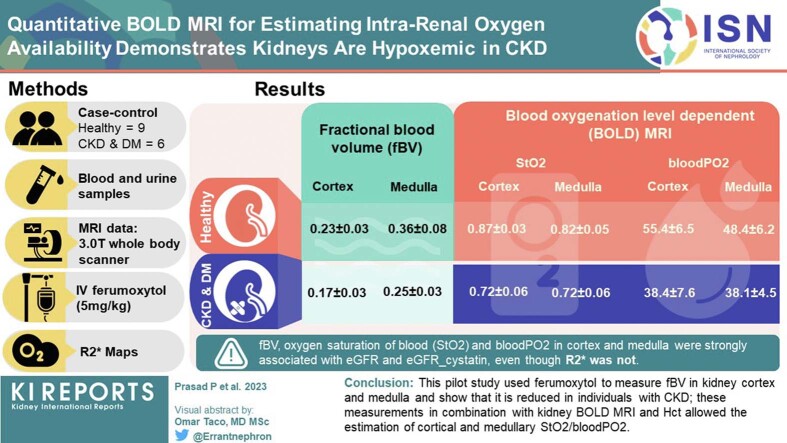

BOLD MRI uses hemoglobin as a reporter of oxygen status and has been shown to be useful in the noninvasive evaluation of relative oxygenation status of the kidneys over 2 decades ago.^1^ The technique is specifically efficacious for monitoring response to acute maneuvers, such as administration of furosemide,^2^ vasomodulators,^3^^,^^4^ waterloading,^5^ etc. MRI acquires images representing a slice through the body and displays the digital images made up of a matrix of numbers, typically 256 × 256. Each of these pixels (picture elements) on the image is actually a representation of 3-dimensional space known as voxel (e.g., 1 mm × 1 mm × 5 mm in the body). BOLD MRI contrast is inherently sensitive to the amount of deoxyhemoglobin within each voxel,^6^^,^S3 which in turn is determined by the fBV, that is, fraction of tissue made up of blood, hematocrit (Hct) or fraction of the blood made up of red blood cells*,* and how well the hemoglobin is oxygenated or oxygen saturation of hemoglobin (StO_2_). When studying acute responses such as following the administration of loop diuretics, it is assumed that there is minimal effect on fBV and Hct, and therefore any observed response was ascribed to changes in oxygen availability, StO_2_. Loop diuretics acutely increase tissue oxygenation by reducing oxygen-consuming tubular reabsorption of sodium.^7^ Over the last 2 decades, kidney BOLD MRI has gained acceptance with validation against independent measures in preclinical models^8^, ^9^, ^10^ and its use in clinical research,^11^, ^12^, ^13^, ^14^, ^15^, ^16^ including in a multicenter setting.^17^ Specifically in animal models, trends observed with BOLD MRI have been compared with those with invasive microprobes, that is, increasing R2∗ was shown to be associated with decreasing oxygenation.^8^, ^9^, ^10^ Early clinical applications of kidney BOLD MRI have been evaluated in patients with renal artery stenosis^11^^,^^15^ and kidney transplantation.^14^^,^^16^

Around the same time as kidney BOLD MRI was gaining initial interest, chronic hypoxia hypothesis^18^ was proposed, which suggests that progressive CKD is associated with chronic hypoxia in the kidney. This led to an interest in extending BOLD MRI to evaluating relative oxygen availability in individuals with CKD.S4 However, an early study indicated that kidney BOLD MRI was not associated with eGFR, a measure of disease severity.^19^ One of the associated letters to the editor^20^ did suggest a potential limitation of not including fBV. Given that chronic hypoxia hypothesis specifically relates hypoxia with disease progression, later studies looked for association with loss of renal function. Three sufficiently large studies published from different countries showed evidence for higher cortical R2∗ (BOLD MRI parameter suggesting lower oxygen availability) to be associated with annual loss of eGFR^21^, ^22^, ^23^ (Please see Supplementary Material for basic understanding of R2∗ and its measurement [Figure S1 For an in-depth understanding refer to Supplementary reference).S1 A smaller but multiparametric study failed to observe a similar observation with cortical R2∗, but indicated that the response to furosemide on medullary R2∗ to be associated with annual loss of renal function.^24^ The first of these reports was accompanied by an editorial comment,^25^ indicating that though the results were in agreement with the chronic hypoxia hypothesis, it was not yet feasible to determine if the kidneys indeed were more hypoxic or hypoxemic in CKD. This is because R2∗ is not specific to StO_2_ but also depends on fBV and Hct.^6^^,^^20^ Because anemia is commonly associated with progressive CKD,^26^ it may confound R2∗ measurements. Data on fBV in humans is lacking, but kidney blood flow is known to be reduced in CKD, given the strong association between kidney blood flow and kidney function.^27^ Perfusion MRI has shown data consistent with this association.^28^ Therefore, it is fair to suspect that fBV may be reduced in CKD. The effects of reduced fBV and Hct have an opposite effect on R2∗ compared to reduced StO_2_, that is, whereas reduced fBV and Hct would decrease R2∗, reduction in StO_2_ will increase R2∗. Considering that all 3 could be reduced in CKD, the net effect on R2∗ may be compromised leading to minimal change in R2∗ observed with disease severity. This was articulated in the editorial comment^25^ and inspired us to undertake the current study.

Quantitative BOLD (qBOLD) MRI methods have been applied to the brain.^29^, ^30^, ^31^ Here, an independent measure of fBV and Hct along with measurements of R2∗ and R2, the inherent spin-spin relaxation rate measured using spin echo MRI, were used to estimate StO_2_ in brain tissue in animal models^29^^,^^30^^,^S5^,^S9 and humans.^31^ fBV can be measured using blood pool contrast agents such as ferumoxytol.^32^^,^^33^^,^S6 Ferumoxytol, because of its physical size stays within the blood pool for an extended period (blood half-life in humans ∼14 hours), and has been used as MRI contrast agent on an off-label basis in the United States. This method was recently applied to rat kidneys and demonstrated an agreement between both fBV and cortical StO_2_ estimates with those from Near Infrared Spectroscopy.^34^ Using Hill’s equationS10 that describes the hemoglobin oxygen desaturation curve, one can also estimate the bloodPO_2_.^35^ Either StO_2_ or bloodPO_2_ can be used to infer the regional oxygen availability. In this pilot case-control study, we have adapted the method previously used in rat brains^29^^,^^30^ and kidneys^34^ to human kidneys and have demonstrated the feasibility of estimating StO_2_ and bloodPO_2_ in the kidney cortex and medulla in a small number of healthy volunteers and individuals with CKD. We used ferumoxytol to measure fBV and used a measure of Hct in peripheral blood to estimate cortical and medullary Hct based on prior literature.^36^ We also availed the opportunity to evaluate whether furosemide has any direct effect on fBV.

## Methods

### Subjects

All procedures were performed with approval from the institutional review board (EH20-117) and written consent from each of the participants. Fifteen subjects participated in this pilot case-control study, with 9 healthy controls and 6 individuals with CKD and diabetes. CKD patients were from our institutional Nephrology Clinics. The healthy participants were recruited from an established database consisting of participants in prior imaging studies at our center. Among the controls, 1 participant did not complete imaging acquisitions and another 1 had inadequate image quality and therefore were not included in the analysis. All patients with CKD were either stage 3 or 4. One subject was taking ferrous sulfate to treat anemia. One subject had a prior administration of ferumoxytol (at least 2 months before the study).

Inclusion criteria for all participants included, age ≥18 years; ability to give informed consent; ability and willingness to follow study protocol; and absence of contraindication for magnetic resonance study including claustrophobia, presence of a pacemaker, intracranial clips, or intraocular debris. Healthy participants for control group should have no history of diabetes, hypertension, or heart disease; be normotensive, that is, <130 mm Hg systolic blood pressure and <90 mm Hg diastolic blood pressure; normal kidney function for age as evidence by GFR >60 ml/min per 1.73 m^2^ calculated by CKD-Epidemiology Collaboration formula. CKD patients should have, a diagnosis of type 1 or type 2 diabetes and 15 ml/min per 1.73 m^2^ < GFR < 60 ml/min per 1.73 m^2^ calculated by CKD-Epidemiology Collaboration formula.

Exclusion criteria for all participants included the following: pregnant or nursing females; history of decompensated heart failure (acute or chronic diastolic or congestive heart failure); history of unilateral kidney disease such as renal artery stenosis or ureteral obstruction; and chronic use of nonsteroidal anti-inflammatory agents; iron overload, defined as serum ferritin >800 ng/ml. CKD patients should not have CKD because of primary glomerular disease, primary interstitial disease, or polycystic kidney disease

Blood specimens were collected for each participant at the screening visit for kidney function before scheduling of MRI scan. Urine samples for protein-to-creatinine ratio were taken immediately before the MRI scan. Participants’ demographic information, including eGFR estimates based on both creatinine and cystatin C using CKD-Epidemiology Collaboration equations are summarized in Table 1.

**Table 1:** Baseline demographic characteristics of study groups

Variable	CKD (*n* = 6)	Control (*n* = 7)
Age, yr	68.4 ± 6.6	40.1 ± 15.3
Gender, male, *n* (%)	2 (33.3%)	2 (28.6%)
White, *n*	3	3
African American, *n*	2	1
Other, *n*	1	3
BMI, kg/m^2^	30.5 ± 3.3	26.1 ± 7.7
Systolic BP, mm Hg	138.8 ± 30.0	109.1 ± 21.7
Hematocrit %	33.2 ± 3.3	40.6 ± 3.5
eGFR creatinine, ml/min per 1.73 m^2^	31.7 ± 13.3	97.9 ± 15.7
eGFR cystatin C, ml/min per 1.73 m^2^	34.3 ± 20.8	114.3 ± 16.2
Alb/creat^a^ mg/g	1033.2 (264.4∼2498.2)	4.0 (3.5∼7.3)

Alb/create, albumin-to-creatinine ratio; BMI, body mass index; BP, blood pressure; eGFR creatinine, estimated glomerular filtration rate based on creatinine levels; eGFR cystatin C, estimated glomerular filtration rate based on cystatin C levels.

Most values shown as mean ± SD.

a Shown as median (interquartile interval).

### MRI Methods

Participants were instructed to fast overnight before coming for the MRI scans performed in the morning. They were also asked to refrain from using nonsteroidal anti-inflammatory drugs for 3 days before the scheduled MRI scans. If they are using insulin, participants were instructed to use half their dose the previous evening and hold the morning dose until after the scan. Similarly, if they were taking blood pressure medications such as angiotensin converting enzyme inhibitors or angiotensin receptor blockers, they were asked to not take the dose on the previous day and hold the scan day’s dose until after the scan. Participants on furosemide were asked to not take the day’s dose.

All MRI data were acquired on a 3.0 T whole body scanner (Magnetom Skyra^Fit^, Siemens Healthcare, Erlangen, Germany). Because R2∗ comprises of 2 primary components, namely, inherent spin-spin relaxation rate R2 and a component related to the susceptibility effect,^37^ qBOLD MRI data include both R2 and R2∗ measurements. Difference in R2∗ and R2 is a component that is specific to susceptibility effects such as because of deoxyhemoglobin or ferumoxytol. R2∗ mappingS2 data were acquired using breath-hold multiple gradient echo sequence and R2 mapping data were acquired with a breath-hold multiple TE turbo spin echo sequence (Table 2).

**Table 2:** Listing of key MRI acquisition parameters for R2∗ and R2 mapping at 3 T

Parameter	R2∗ map_pre	R2∗ map_post	R2_map
Sequence	Multiple Gradient Echo	Multiple Gradient Echo	Multiple Spin Echo
Field of view	400 mm	400 mm	400 mm
Slice thickness	2 mm	2 mm	5 mm
Repetition time	60 ms	40 ms	500 ms
Echo time	4.92, 9.84, …, 39.36	2.36, 3.55, …, 10.69	7.8, 39, 70, …, 194
Average	1	1	1
Flip angle	30°	30°	90°
Matrix	192 × 192	192 × 192	256 × 256
# of slices	3	3	3
# of echos	8	8	7
Bandwidth	280 Hz/pixel	1085 Hz/pixel	465 Hz/pixel
Slice interval	5 mm	5 mm	5 mm

Given the magnitude of change in R2∗ post-ferumoxytol (5 mg/kg), we used shorter echo times.

**Figure 1:**
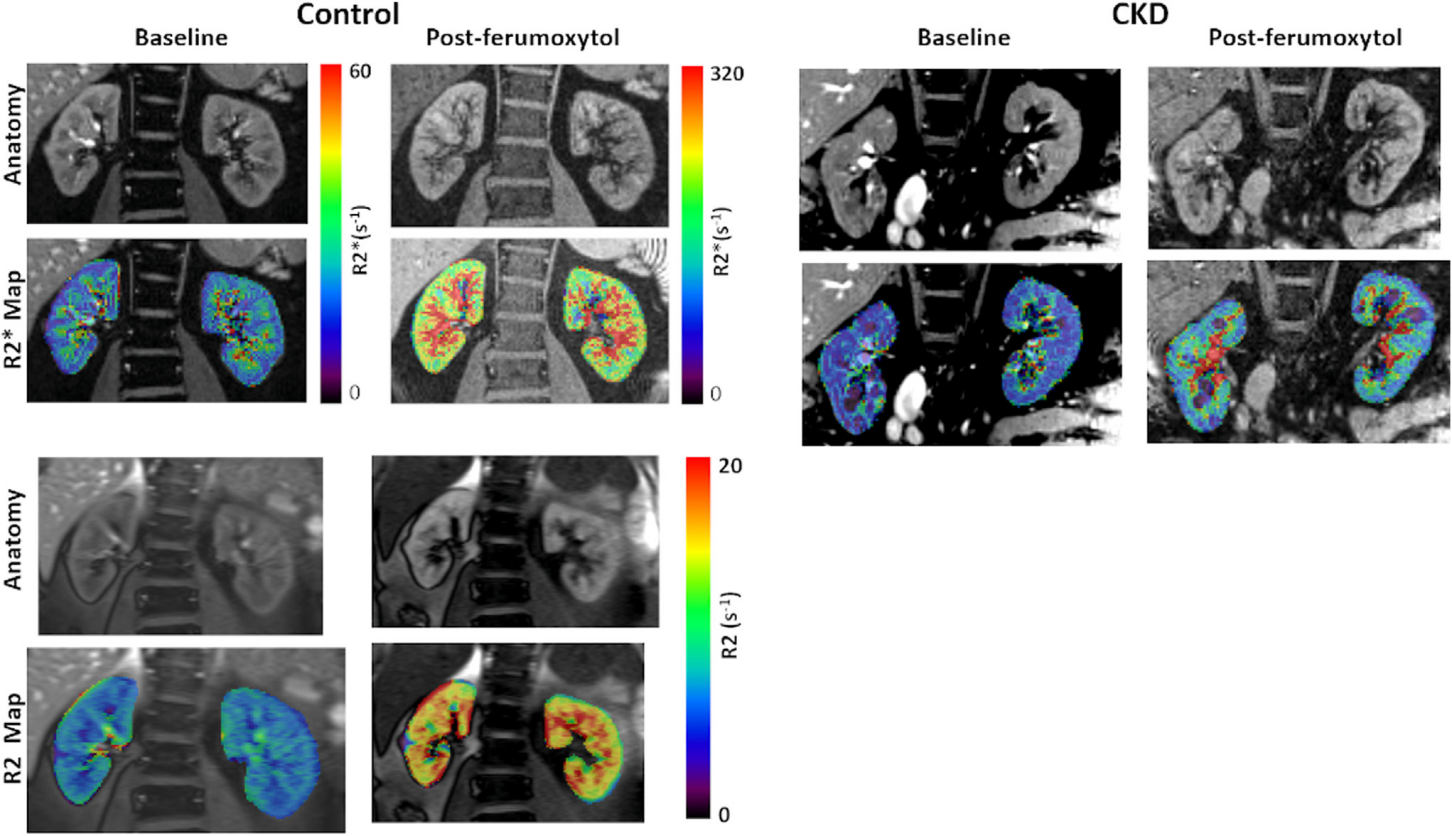
R2∗ and R2 maps acquired at baseline and post-ferumoxytol (5 mg/kg) in a representative control (eGFR 77) (Left) and individual with CKD (eGFR 23) (Top Right). The R2∗ maps for control and CKD used the same color bar. Note the substantial increase in R2∗ following ferumoxytol both in the cortex and medulla, but much smaller increase in the individual with CKD, suggesting reduced fBV. Though R2 also shows a similar trend of increase (Bottom), the magnitude of change is much smaller. Both R2∗ and R2 show a higher degree of enhancement in the medulla compared to the cortex, suggesting higher fBV in the medulla. CKD, chronic kidney disease.

After baseline R2∗ images were acquired using the multiple gradient echo sequence (R2∗ Map_pre in Table 2), ferumoxytol (5 mg/kg) was administered intravenously using a Medrad Spectris (Bayer Healthcare, NJ) MRI compatible power injector. The dose was diluted to 100 ml in saline and was administered at a rate of 0.1 ml/s over approximately 17 minutes, consistent with standard recommendations.^38^ Blood pressure was monitored at 5 minute intervals during the administration and MRI scan using Invivo 3150 (Phillips Healthcare, Andover, MA) MRI patient monitor. Post-ferumoxytol R2∗ maps were acquired with the same multiple gradient echo sequence but using a higher bandwidth to support shorter echo times (R2∗ Map_post in Table 2). Representative R2∗ Maps obtained before and after administration of ferumoxytol are shown in Figure 1. Data were also acquired following an administration of furosemide (0.5 mg/kg) with a maximum dose of 40 mg. The post furosemide R2∗ scans were performed up to 20 minutes or until the participant had to urinate. Only 5 of the 7 in the control group received furosemide because of logistical constraints.

### Regions of Interest Analysis

**Figure 2:**
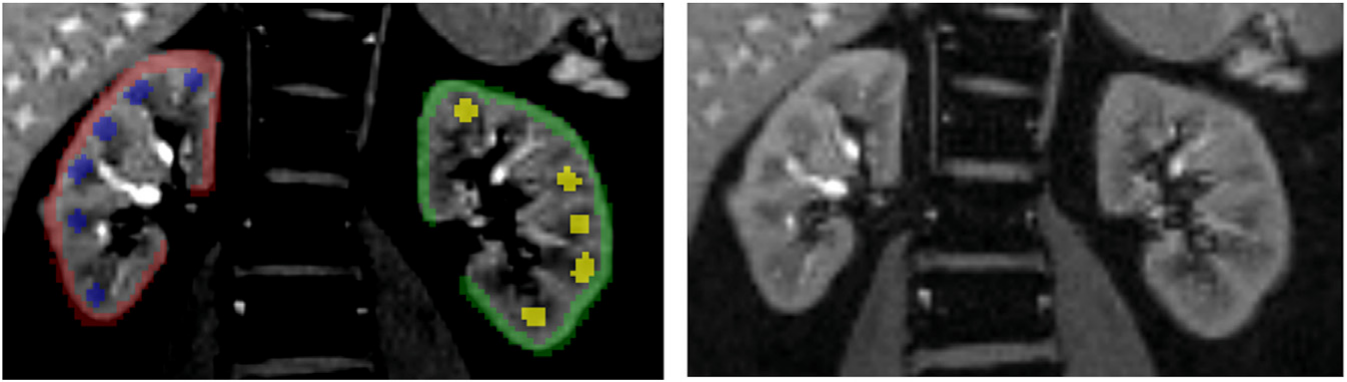
Illustration of manually defined regions of interest (ROI) in the cortex and medulla (Left). (Right) shows same image without overlaid ROIs. FireVoxel considers all the ROIs of same color to be a single ROI whether or not the pixels are all connected. For the defined ROIs, we get an output of 4 measures: Left_cortex, Left_medulla, Right_cortex, and Right_medulla. We combine Left and Right values for each region per participant.

The image analysis was performed using FireVoxel (firevoxel.org), a noncommercial research-only software freely distributed by the developers at New York University. Manual definition of regions of interest in kidney cortex and medulla were drawn for each slice on each kidney (Figure 2). FireVoxel performs quantitative parametric mapping and then reports regional R2∗ value within each region for each kidney. Data from left and right kidneys were combined to define 1 representative value for each region per participant.

Using the pre-ferumoxytol and post-ferumoxytol regional R2∗ values, we estimated regional fBV. Then using regional fBV values along with estimated regional Hct (based on measurement in peripheral blood),^36^ we estimated regional StO_2_ and blood PO_2_ (Please see Supplementary Material for technical details and specific equations used). For more in-depth understanding use supplementary reference.S8

### Statistical Analysis

Given the small sample size, for comparing control versus CKD, nonparametric Mann-Whitney U test was performed. Associations of MRI estimates with kidney function were performed using Spearman correlation. Pair-wise comparisons were performed using nonparametric Wilcoxon test for evaluating response to furosemide. All statistical analysis were performed using SPSS 22.0 software (IBM Corp., Armonk, NY). *P* < 0.05 was considered as statistically significant. In case of missing data, the specific participants were excluded from the analysis.

## Results

The demographic data for the participants (7 controls and 6 with CKD) in the study whose MRI data are reported are summarized in Table 1.

**Figure 3:**
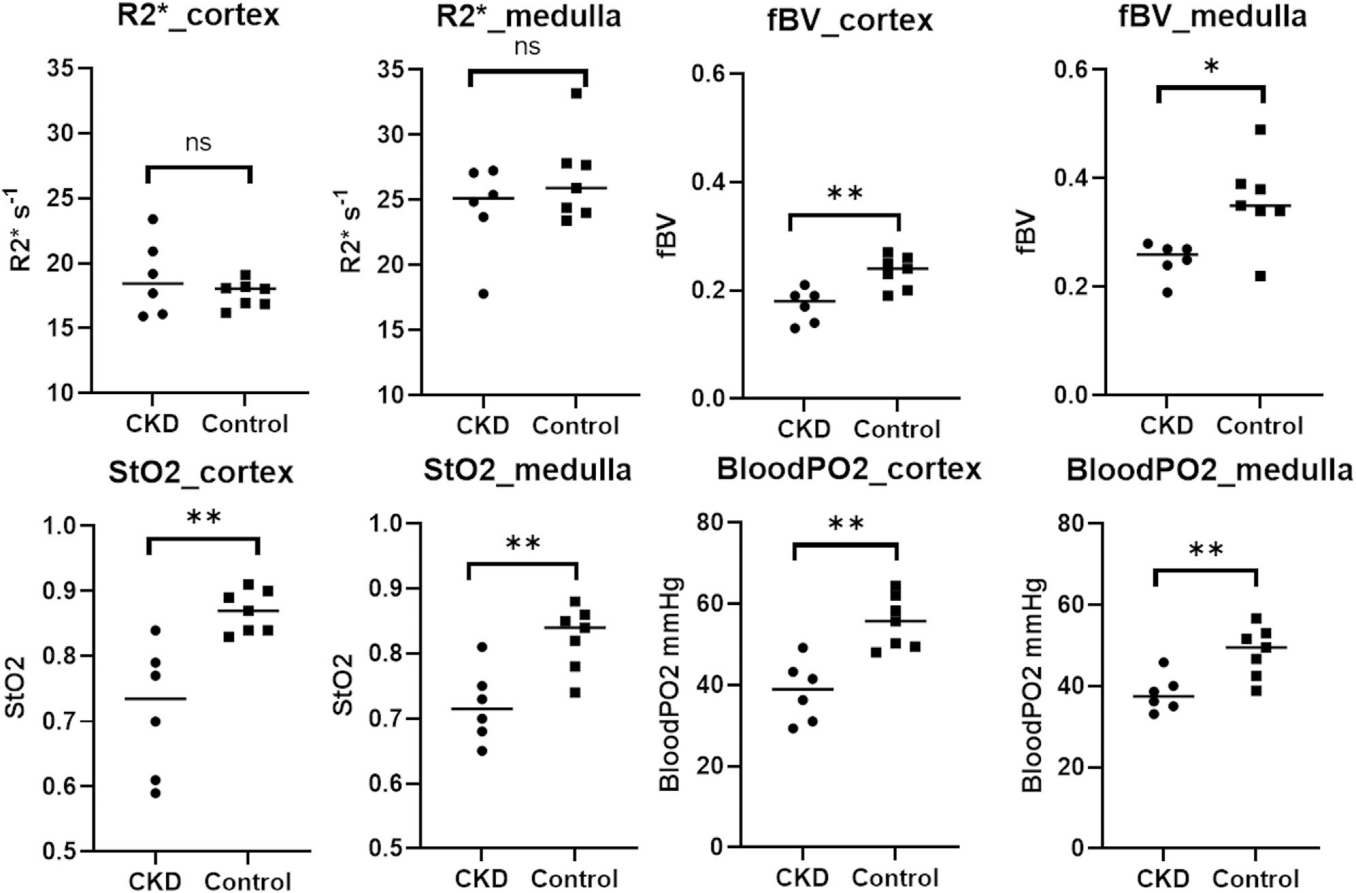
Individual scatter plots summarizing R2∗, fBV, StO_2_, and blood pO_2_ in both the cortex and medulla obtained in the 2 groups of individuals participated in the study (CKD vs. Control). Note the significantly lower values in CKD for fBV, StO_2_, and blood pO_2_ whereas R2∗ shows only minimal differences. Further, R2∗ in the medulla is lower in CKD, which may be wrongly interpreted as the oxygenation to be improved in CKD. ∗*P* < 0.05 and ∗∗*P* < 0.01 by nonparametric Mann-Whitney test. CKD, chronic kidney disease; fBV, fractional blood volume.

R2∗ maps at baseline and post 5 mg/kg of ferumoxytol in a representative control participant and an individual with CKD are shown in Figure 1. In the presence of ferumoxytol, R2∗ values increase substantially, however the relative level of enhancement was lower in CKD. These changes were used to estimate regional fBV using equation S1. Using this estimate along with baseline regional R2∗ and R2 values, estimates of regional StO_2_ and bloodPO_2_ were obtained, using equation S3 and S4, respectively. These measurements are summarized in Figure 3 and Table 3 for the 2 groups of participants. Both estimated StO_2_ and bloodPO_2_ were significantly lower in CKD in both kidney cortex and medulla, even though the corresponding R2∗ values were not significantly different. Consistent with our prior reports, the median medullary R2∗ in CKD was slightly lower than that in healthy controls.

**Table 3:** Group comparison of blood oxygenation level dependent magnetic resonance imaging parameters between the 2 groups of participants

Measurements	Participant	N	Mean	SD	Significance *P*-value
R2∗_cortex s^−1^	Control	7	17.64	1.00	0.700
	CKD	6	18.88	2.92	
R2∗_medulla s^−1^	Control	7	26.64	3.37	0.170
	CKD	6	24.36	3.49	
fBV_cortex	Control	7	0.23	0.03	0.005
	CKD	6	0.17	0.03	
fBV_medulla	Control	7	0.36	0.08	0.022
	CKD	6	0.25	0.03	
StO_2__cortex	Control	7	0.87	0.03	0.005
	CKD	6	0.72	0.10	
StO_2__medulla	Control	7	0.82	0.05	0.008
	CKD	6	0.72	0.06	
Blood PO_2__cortex mm of Hg	Control	7	55.44	6.47	0.002
	CKD	6	38.42	7.62	
Blood PO_2__medulla mm of Hg	Control	7	48.41	6.20	0.008
	CKD	6	38.13	4.49	

bloodPO_2_, oxygen partial pressure of blood; fBV, fractional blood volume; StO_2_, oxygen saturation of hemoglobin.

R2∗, apparent spin-spin relaxation rate measured in the presence of magnetic susceptibility differences measured using gradient echo MRI.

Significance by Mann-Whitney U test.

The Spearman correlation coefficients for regional fBV, StO_2_, and bloodPO_2_ with eGFR_creatinine and eGFR_cystatin-C, as well as urine albumin-to-creatinine ratio are summarized in Table 4. fBV, StO_2_ and bloodPO_2_ in the cortex and medulla were strongly associated with eGFR_creatinine and eGFR_cystatin, even though R2∗ was not. Only cortical fBV, StO_2_, and bloodPO_2_ were associated with urine albumin-to-creatinine ratio.

**Table 4:** Associations (by Spearman correlations) of blood oxygenation level dependent magnetic resonance imaging parameters in cortex (cor) and medulla (med) with disease severity as assessed by renal function (eGFR, UACR)

Renal function		R2∗_cor	R2∗_med	fBV_cor	fBV_med	StO_2__cor	StO_2__med	bPO_2__cor	bPO_2__med
eGFR_creatinine	Spearman ρ	−0.160	0.473	0.758^b^	0.683^a^	0.766^b^	0.583^a^	0.786^b^	0.577^a^
	Sig. (2-tailed)	0.603	0.102	0.003	0.010	0.002	0.036	0.001	0.039
	N	13	13	13	13	13	13	13	13
UACR	Spearman ρ	0.061	−0.452	−0.659^a^	−0.486	−0.655^a^	−0.375	−0.657^a^	−0.38
	Sig. (2-tailed)	0.844	0.121	0.014	0.093	0.015	0.207	0.015	0.201
	N	13	13	13	13	13	13	13	13
eGFR_cystatin C	Spearman ρ	−0.330	0.248	0.887^b^	0.876^b^	0.909^b^	0.869^b^	0.912^b^	0.863^b^
	Sig. (2-tailed)	0.271	0.415	5.28E-05	8.61E-05	1.67E-05	1.14E-04	1.40E-05	1.47E-04
	N	13	13	13	13	13	13	13	13

bPO_2_ refers, to bloodPO_2_ (oxygen partial pressure of blood); eGFR_creatinine, estimated glomerular filtration rate based on creatinine levels; eGFR_cystatin C, estimated glomerular filtration rate based on cystatin C levels; fBV, fractional blood volume; StO_2_, oxygen saturation of hemoglobin; UACR, urine albumin-creatinine ratio.

R2∗, apparent spin-spin relaxation rate measured in the presence of magnetic susceptibility differences measured using gradient echo MRI.

a Correlation is significant at the 0.05 level (2-tailed).

b Correlation is significant at the 0.01 level (2-tailed).

**Figure 4:**
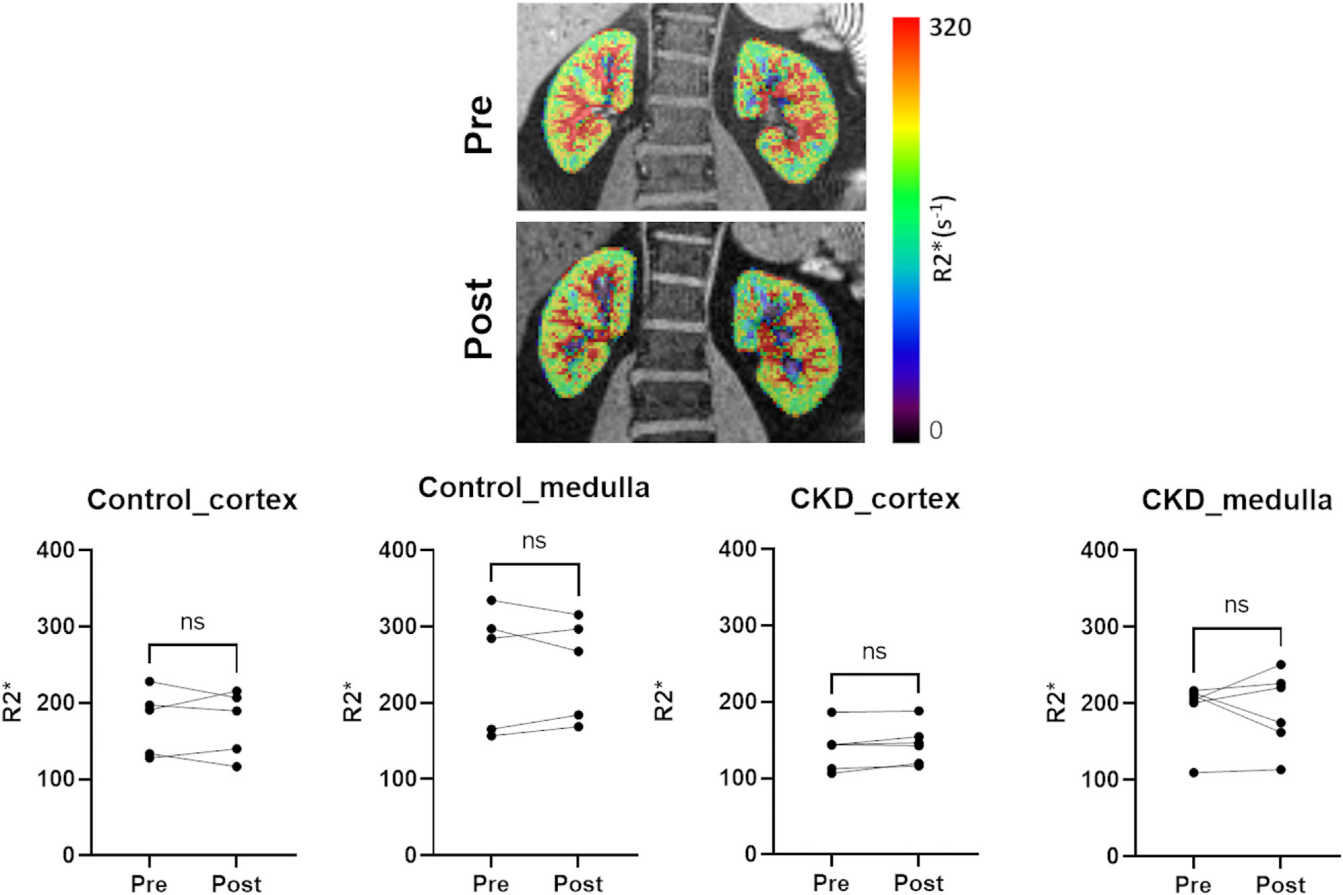
Post-ferumoxytol R2∗ maps acquired pre-furosemide and post-furosemide (0.5 mg/kg) with maximum dose of 40 mg (Top). Plots showing individual responses to furosemide in both the cortex and medulla in each of the 2 groups of participants (Bottom). Note the minimal change in median R2∗ post-furosemide in either group of participants. CKD, chronic kidney disease.

The administration of furosemide does not appreciably change the mean post-ferumoxytol R2∗ but demonstrates variable response in the medulla in terms of directionality (i.e., higher or lower), although the magnitude of change was relatively minimal (Figure 4).

## Discussion

The data presented here reports for the first time fBV in human kidneys, which is significantly decreased in individuals with CKD. Most interestingly, taking fBV into account, the estimated StO_2_ and bloodPO_2_ indicate that kidney cortex is normoxemic in healthy controls but moderately hypoxemic in CKD. Similarly, kidney medulla is mildly hypoxemic in controls but moderately hypoxemic in CKD. These estimates are quite consistent with the chronic hypoxia hypothesis, a fact that has remained elusive. To-date, the only data supporting a possible existence of hypoxia in human kidneys are based on histologic assessments of hypoxia inducible factor activation.^39^ More importantly, qBOLD data clearly show that both the cortex and medulla are more hypoxemic in CKD, whereas the differences observed on uncorrected R2∗ would have been interpreted as an anomalous increase in PO_2_ in the medulla in CKD. A prior report showed that the reduced medullary R2∗ was associated with annual loss of kidney function.^40^

The main conclusion to be drawn from Table 4 is that qBOLD parameters in the cortex are associated with disease severity whereas BOLD MRI parameter R2∗ was not. Note that the associations were not significant when only individuals with CKD were included in the analysis (data not shown). It is not yet clear whether eGFR_Cystatin-C is a better marker of severity of CKD, given the stronger and potentially more significant associations.

How do our estimates of fBV and bloodPO_2_ compare with literature values?

There is very limited literature on kidney fBV. Our data suggest substantially higher fBV in the medulla compared to the cortex. Even though this is apparently counterintuitive, there are prior data to support this observation. A recent report using an enhanced microfill technique^41^ visually demonstrated that kidney medulla has relatively higher fBV compared to that in the cortex. A study from 1970s using labeled blood cells and plasma reported that in rat kidneys, the medulla has higher blood volume than the cortex.^36^ Recent preclinical MRI data using ferumoxytol also indicated higher fBV in the medulla than in the cortex.^34^ Prior preclinical MRI data also demonstrated increased fBV post-ferumoxytol,^42^ using a similar nano particular iron oxide contrast media.^43^ In fact, the latter report demonstrated higher enhancement in the inner medulla, some of which may be related to increased concentration of the agent in inner medulla related to reduced Hct.^36^ Note that the medullary regions of interest used in this study (Figure 2) represent the outer medulla, consistent with prior reports.^44^^,^^45^

Measurements by labeled blood cells and labeled albumin in young healthy volunteers reported cortical vascular volumes in the range of 37 to 57 ml/100 g.^46^ Fractional moving blood estimates by Doppler ultrasound reported value of 0.20 in a single healthy volunteer under 6-hour fasting condition and 0.29 after hydration with 1.4 L of water.^47^ One prior report using an agent similar to ferumoxytol and dynamic MRI to follow first pass kinetics also showed data suggesting higher fBV in the medulla compared to the cortex.^48^ This article also reported cortical fBV of 0.41. Our reported value of 0.23±0.03 in controls is comparable to values reported by Doppler ultrasound.

Although a number of reports demonstrated the measurement of tissue oxygenation using microelectrodes or probes in preclinical models^7^, ^8^, ^9^, ^10^, we know of only 1 report on the measurement of vascular PO_2_.^49^ Using 2 different oxyphors selectively within the vascular and tissue compartments of rat kidneys,^50^ the authors measured PO_2_ in both compartments simultaneously. Our estimates of bloodPO_2_ (Table 3) in healthy human kidneys are in agreement with this report. Note that our MRI derived measurements include both arterial and venous vasculature within each voxel. Zhang *et al.*^51^ had previously proposed a numerical method based on Monte Carlo simulations to relate measured R2’ (i.e., R2∗ − R2) with an estimate of StO_2_. However, the report did not include an independent measure of fBV or Hct and used “typical values” of 40% and 25% for fBV and 0.4 and 0.2 for Hct in cortex and medulla, respectively. In healthy volunteers, the cortical blood PO_2_ was estimated to be 58 mm Hg and StO_2_ of 0.92, quite comparable to our estimates. The only reported direct measures of tissue PO_2_ in human kidneys^52^ using microelectrodes in individuals undergoing nephrectomy (*n* = 2) show values in the range of 88 to 100 mm Hg in the cortex, 38 to 68 mm Hg in the medulla and 18 to 26 mm Hg in inner medulla. Our estimated values in the medulla are consistent with the range in the outer medulla.

A recent report based on preclinical application of the method used here indicated a potential for overestimation of fBV in the medulla because of contributions of large vessels outside the slice in the vicinity of medulla.^34^ However, they also demonstrated that fBV was higher in the medulla based on alternate contrast mechanism R2, which is resistant to bulk susceptibility effects. Similarly, we have seen increased R2 values in the medulla post-ferumoxytol (Figure 1). We also attempted to mitigate the artifactual contributions because of external macroscopic susceptibility sources on R2∗ maps by using 2 mm slices (instead of 5 mm commonly used in most of the prior reports on kidney BOLD MRI). Because the post-ferumoxytol changes in R2 was much smaller compared to that in R2∗, the use of R2 in the estimation of fBV is not indicated.

Overall, our data demonstrate the feasibility of noninvasive quantitative estimation of bloodPO_2_ within the human kidney. Availability of StO_2_ or bloodPO_2_ can facilitate classification of relative regional hypoxemia. In this regard, this is the first demonstration of kidney hypoxemia in human kidneys using a noninvasive measure that can be translated to the clinic. Even though, the method necessitates the off-label use of an approved agent, ferumoxytol is compatible and may be preferred for use in individuals with CKD.^53^^,^^54^ It should be noted that ferumoxytol was originally developed as an MRI contrast agent,^55^ but is currently approved for use as iron replacement in the US,^56^ and there is growing interest in its use in MRI primarily for vascular imaging in those for whom gadolinium is contraindicated.^38^

Considering that furosemide did not demonstrate consistent changes in post-ferumoxytol R2∗ in both the cortex and medulla, there is no indication that fBV is reduced post-furosemide. The variable response observed in the medulla is most probably related to partial volume effects. Prior preclinical reports have indicated a transient decrease in R2∗ in the inner medulla following furosemide after administration of ultrasmall superparamagnetic iron oxide with minimal changes in the cortex and outer medulla.^43^ This further supports the view that the widely verified observation of decreased medullary R2∗ (without ferumoxytol) following furosemide^2^ is primarily determined by the improved StO_2_ related to reduction in oxygen consumption consistent with prior microelectrode data in rat kidneys.^7^

All clinical applications pursued with BOLD MRI should be feasible with qBOLD MRI. Apart from CKD, we believe another clinical application of qBOLD MRI is in the evaluation of renal artery stenosis. Given that fBV may be reduced because of the stenosis, the measured kidney R2∗ values do no indicate the true levels of hypoxemia. Although measurements using an intravascular agent may be preferred for fBV measurements, alternate methods such as using conventional gadolinium agents^57^ or using intravascular incoherent motion,^58^ which is a noncontrast method, are available to estimate fBV. Future studies are necessary to evaluate the level of agreement between the different estimates.

Our study is not without limitations. Ferumoxytol is approved for human use in the US and is being used both as a therapeutic and/or diagnostic agent, but is not being used in other countries. This preliminary feasibility study included only a small number of participants and the groups were not age matched. However, it should be mentioned that even with such small sample size, the data demonstrated statistically significant differences between controls and CKD. Multiple Spearman’s rank analysis increase the risk of multiple tests, without Bonferroni correction. Given the preliminary nature of this report with a limited sample size, we did not perform the correction. Our analysis did not include pH and other variables that could potentially affect the hemoglobin oxygen desaturation curve. We used the measure of Hct in peripheral blood to estimate Hct in the cortex and outer medulla (0.9 × Hct of peripheral blood) based on prior preclinical data.^36^ Future studies should evaluate R2∗ versus dose of ferumoxytol to optimize the dose and improve spatial resolution that provides adequate sensitivity but limits the artifactual contributions. We also contend+ that future studies should use ferumoxytol dose based on total blood volume estimate based on Nadler’s equation^59^^,^S7 rather than simply by body weight. This could improve the similarity of R2∗ values post-ferumoxytol across participants. Future studies should also consider comparing the MRI derived measurements with independent measures of fBV and/or bloodPO_2_. In this preliminary report, we only showed regions of interest measurements, but in the future it should be feasible to create StO_2_ and bloodPO_2_ maps. However, it will require registration of data from different breath-holds.

In conclusion, we present preliminary experience using ferumoxytol to measure fBV in kidney cortex and medulla and show for the first time that it is reduced in individuals with CKD. These measurements in combination with kidney BOLD MRI measurements allowed us to estimate cortical and medullary StO_2_ and bloodPO_2_, the values of which are consistent with the limited literature to-date. StO_2_ and bloodPO_2_ were significantly lower in individuals with CKD, both in kidney cortex and medulla, consistent with chronic hypoxia hypothesis. The data in the medulla is significant because currently, based on reduced R2∗ in CKD compared to controls leads to a wrong interpretation that medullary oxygen availability is improved in CKD. Overall, qBOLD MRI allows for direct evaluation of kidney oxygen status and therefore facilitates mechanistic evaluation of interventions targeting kidney hypoxia such as sodium glucose cotransporter inhibitors.^60^ The method as described can be translated to the clinic, however it may require further studies with a larger number of participants.

## Disclosure

All the authors declared no competing interests.

## Supplementary Material

Supplementary File (PDF)

**Basic explanation of R2∗ and technical details.**

**Supplementary References.**

**Figure S1.** The signal observed in MRI is the transverse component that can be picked by a receiver coil and a typical signal evolution in time is illustrated here. A 90° radiofrequency (RF) pulse flips the longitudinal magnetization to the transverse plane. The resulting signal is called the free induction decay (FID). The signal decay caused by microscopic processes depends on intrinsic factors, such as molecular size and tissue type, and occurs on a timescale denoted T2. Dephasing over a larger scale is a result of effects such as magnetic field inhomogeneity, such as due to the presence of iron or deoxygenated hemoglobin. This further shortens the coherence time of the transverse magnetization within a given volume of tissue, to a value denoted T2∗. This decay is irreversible and can be measured by sampling the FID. Application of an 180° pulse reverses the dephasing and results in the formation of an echo at echo time (TE), as illustrated. This is termed a spin-echo and the signal loss compared to time zero can be used to measure T2.
