# Supplementary material for: Quantitative Blood Oxygenation Level Dependent Magnetic Resonance Imaging for Estimating Intra-renal Oxygen Availability Demonstrates Kidneys Are Hypoxemic in Human CKD
Source: Kidney Int Rep. 2023 Mar 7;8(5):1057–67. doi: 10.1016/j.ekir.2023.02.1092 (PMC10166744; doi:10.1016/j.ekir.2023.02.1092)
Supplement: Supplementary File (PDF) [file mmc1.docx]

**Supplementary Material:**

**Basic explanation of R2*:**

Signal in magnetic resonance imaging (MRI) originates from the nuclei within water molecules, which are associated with magnetic properties. However, in nature these individual water nuclei are randomly oriented due to thermodynamics, resulting in no net magnetic field. When placed in a strong external magnet (typically ten thousand times the earth’s magnetic field), the nuclei become aligned either along or against the field with a slightly higher number aligned with the external field. This results in a net magnetic longitudinal magnetization. The individual nuclei precess along the external applied field, but with random phases resulting in a zero net transverse magnetization. The longitudinal magnetization can be perturbed by an external radio frequency (RF) pulse applied at the resonant frequency (a function of the external magnetic field). Once the external RF pulse is turned OFF, the nuclei return to the equilibrium determined by two fundamental constant T1 (longitudinal) and T2 (transverse) relaxation times (or R1(1/T1) and R2 (1/T2) relaxation rates). Much of clinical MRI is based on these two time/rate constants to create contrast in images. Figure S1 illustrates the T2 and T2* decay and how they can be measured using spin echo or free induction decay respectively.


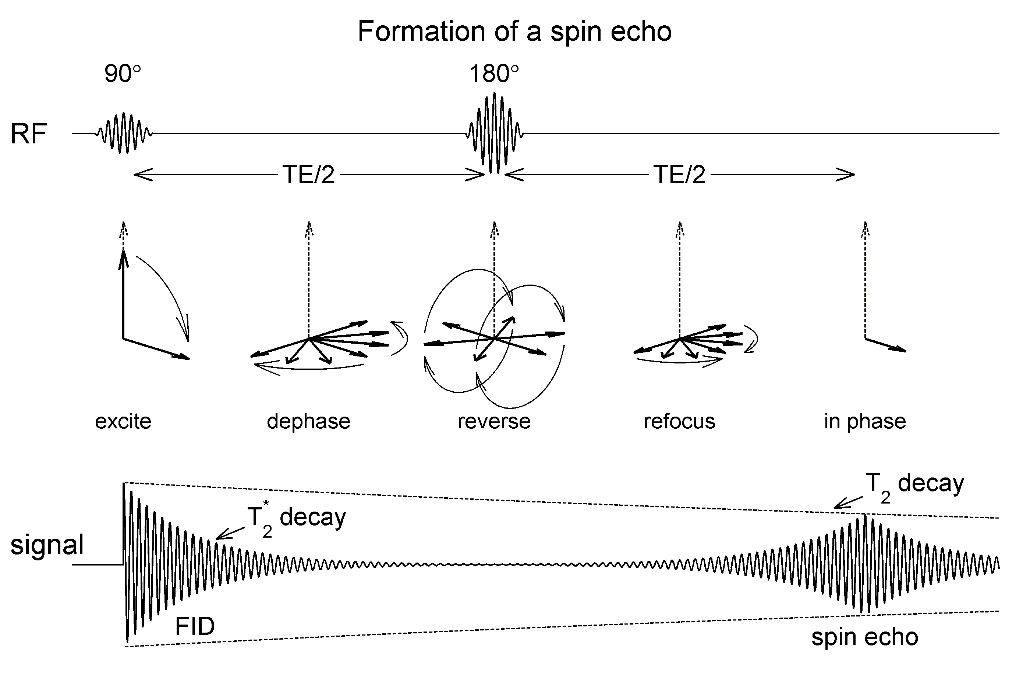


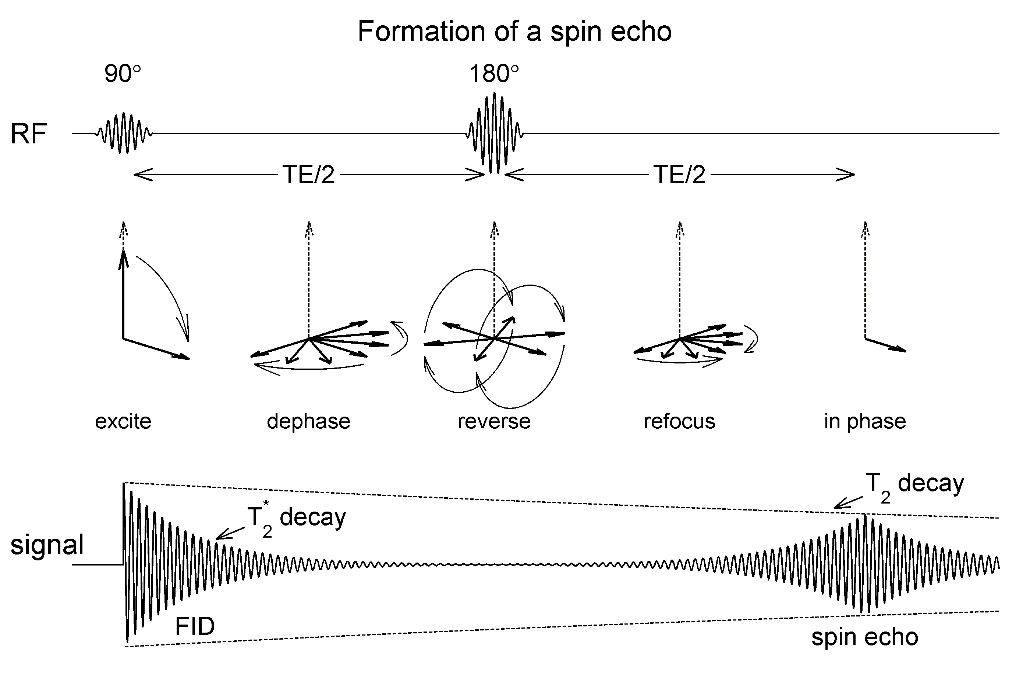


**Supplementary Figure S1:** The signal observed in MRI is the transverse component that can be picked by a receiver coil and a typical signal evolution in time is illustrated here. A 90^o^ degree radiofrequency (RF) pulse flips the longitudinal magnetization to the transverse plane. The resulting signal is called the free induction decay (FID). The signal decay caused by microscopic processes depends on intrinsic factors, such as molecular size and tissue type, and occurs on a timescale denoted T2. Dephasing over a larger scale is a result of effects such as magnetic field inhomogeneity, such as due to the presence of iron or deoxygenated hemoglobin. This further shortens the coherence time of the transverse magnetization within a given volume of tissue, to a value denoted T2*. This decay is irreversible and can be measured by sampling the FID. Application of an 180^o^ pulse reverses the dephasing and results in the formation of an echo at echo time (TE), as illustrated. This is termed a spin-echo and the signal loss compared to time zero can be used to measure T2. For more in-depth understanding please refer^1^.

T2*/R2* mapping has become the standard for imaging iron deposition in the liver, kidneys, heart *etc*. in patients with thalassemia, hemochromatosis and sickle cell anemia^2^. Since deoxygenated hemoglobin has magnetic properties, R2* mapping is also useful to map oxygen availability within tissue and is generally known as blood oxygenation level dependent (BOLD) MRI. However, deoxygenated hemoglobin is two orders of magnitude weaker compared to iron in ferumoxytol in terms of magnetic properties and so the relative changes in R2* observed with BOLD MRI are relatively smaller. While commonly used in the brain to spatially map neuronal activity^3^, BOLD MRI was applied to the kidneys in 1990s and since then R2* mapping has shown to be sensitive to changes in different diseases including CKD^4^. The difference in R2* and R2 represents the component sensitive to magnetic inhomogeneity and is determined by how much iron or deoxygenated hemoglobin is present within a unit imaging volume (voxel). Since ferumoxytol behaves as an intravascular agent, the amount of iron within the voxel is governed by fractional blood volume (fBV). For BOLD MRI, the amount of deoxygenated hemoglobin is determined by a combination of fBV, hematocrit and oxygen saturation of hemoglobin.

*Estimation of fBV and StO2*

Using pre- and post-ferumoxytol R2* values in cortex and medulla, regional fBV can be calculated using the following formula^5^:

$$fBV=\frac{3}{4\pi} \frac{\left( {{R2}^{*}}_{ferumoxytol}-{{R2}^{*}}_{baseline} \right)}{\gamma B_{0}\Delta\chi_{ferumoxytol}} eq. S1$$

where γ is the gyromagnetic ratio, B_o_ is the field strength in Tesla and $\Delta\chi_{ferumoxytol}$ is the susceptibility difference due to ferumoxytol and is given by

$$\Delta\chi_{ferumoxytol}= \frac{m_{Fe}}{M_{Fe}TBV}\frac{M_{sat}}{B_{0}}$$

where *M_sat_* is the saturation magnetization of ferumoxytol and is equal to 0.396 μT/mM of iron^6^. *M_Fe_* is the standard atomic weight of iron = 55.85 u, *m_Fe_* is the ferumoxtyol dose, and TBV is the total blood volume estimated based on subjects height and weight using Nadler’s formula^7^.

Using the fBV data, a recent study has estimated StO2 in the brain using following equation^8^: $s\left( t \right)= K.exp\left[ -R2\cdot t-fBV\cdot\gamma.\frac{4\pi}{3}\cdot\Delta\chi_{0}\cdot Hct\cdot\left( 1-StO2 \right)\cdot B_{0}\cdot t \right] eq.S2$

where s(t) is the signal decay of T2* weighted sequence, K is a proportionality constant, Δχ_0_ is the magnetic susceptibility difference between fully oxygenated and fully deoxygenated hemoglobin (0.264 ppm), and B_0_ is the field strength. The validity of the model for estimating fBV and StO2 using eq. 2 was numerically verified previously, especially to test the impact of the underlying assumptions^9^. The MR estimates of fBV were compared to that from two photon microscopy.

The exponential term in eq. 2 represents the R2* and so one could estimate StO2 from a measurement of R2* and R2.

${R2}^{*}= -R2\cdot t-fBV\cdot\gamma\cdot\frac{4\pi}{3}\cdot\Delta\chi_{0}\cdot Hct\cdot\left( 1-StO2 \right)\cdot B_{0}$

$\therefore StO2=1-\left( \frac{3}{4\pi}\frac{\left( {R2}^{*}-R2 \right)}{fBV\cdot\gamma\cdot\Delta\chi_{0}\cdot Hct\cdot B_{0}} \right) eq. S3$

Using Hill’s equation^10^, StO2 can be converted to bloodPO2.

$StO2= \frac{P_{1}^{h}}{P_{1}^{h}+P_{50}^{h}} eq. S4$

where P_1_ is bloodPO2, h ~ 2.55, P_50_ ~ 26 mmHg is the oxygen partial pressure in plasma at StO2 of 0.5.

**Supplementary References:**

1. Storey P. Introduction to magnetic resonance imaging and spectroscopy. *Methods Mol Med.* 2006;124:3-57.

2. Aslan E, Luo JW, Lesage A, et al. MRI-based R2* mapping in patients with suspected or known iron overload. *Abdom Radiol (NY).* 2021;46(6):2505-2515.

3. Roalf DR, Gur RC. Functional brain imaging in neuropsychology over the past 25 years. *Neuropsychology.* 2017;31(8):954-971.

4. Pruijm M, Mendichovszky IA, Liss P, et al. Renal blood oxygenation level-dependent magnetic resonance imaging to measure renal tissue oxygenation: a statement paper and systematic review. *Nephrol Dial Transplant.* 2018;33(suppl_2):ii22-ii28.

5. Christen T, Bouzat P, Pannetier N, et al. Tissue oxygen saturation mapping with magnetic resonance imaging. *J Cereb Blood Flow Metab.* 2014;34(9):1550-1557.

6. Rivera-Rivera LA, Johnson KM, Turski PA, Wieben O, Schubert T. Measurement of microvascular cerebral blood volume changes over the cardiac cycle with ferumoxytol-enhanced T2 (*) MRI. *Magn Reson Med.* 2019;81(6):3588-3598.

7. Nadler SB, Hidalgo JH, Bloch T. Prediction of blood volume in normal human adults. *Surgery.* 1962;51(2):224-232.

8. Yablonskiy DA, Haacke EM. Theory of NMR signal behavior in magnetically inhomogeneous tissues: the static dephasing regime. *Magn Reson Med.* 1994;32(6):749-763.

9. Christen T, Zaharchuk G, Pannetier N, et al. Quantitative MR estimates of blood oxygenation based on T2*: a numerical study of the impact of model assumptions. *Magn Reson Med.* 2012;67(5):1458-1468.

10. Severinghaus JW. Simple, accurate equations for human blood O2 dissociation computations. *Journal of applied physiology: respiratory, environmental and exercise physiology.* 1979;46(3):599-602.
